# Optimal Position and Orientation of an Ossicular Accelerometer for Human Auditory Prostheses

**DOI:** 10.3390/s24248084

**Published:** 2024-12-18

**Authors:** Dmitrii Burovikhin, Panagiota Kitsopoulos, Michael Lauxmann, Karl Grosh

**Affiliations:** 1 Reutlingen Research Institute, Reutlingen University, Alteburgstr. 150, 72762 Reutlingen, Germany; dmitrii.burovikhin@reutlingen-university.de; 2 Department of Mechanical Engineering, University of Michigan, 2350 Hayward St., Ann Arbor, MI 48109, USA; pkitsop@umich.edu; 3 Faculty of Engineering, Reutlingen University, Alteburgstr. 150, 72762 Reutlingen, Germany; michael.lauxmann@reutlingen-university.de; 4 Department of Biomedical Engineering, University of Michigan, 1101 Beal Ave, Ann Arbor, MI 48109, USA

**Keywords:** implantable auditory prostheses, implantable biomedical devices, finite element method, middle ear, cochlear implants, incus

## Abstract

In this study, a method for determining the optimal location and orientation of an implantable piezoelectric accelerometer on the short process of the incus is presented. The accelerometer is intended to be used as a replacement for an external microphone to enable totally implantable auditory prostheses. The optimal orientation of the sensor and the best attachment point are determined based on two criteria—maximum pressure sensitivity sum and minimum loudness level sum. The best location is determined to be near the incudomalleolar joint. We find that the angular orientation of the sensor is critical and provide guidelines on that orientation. The method described in this paper can be used to further optimize the design and performance of the accelerometer.

## 1. Introduction

By 2050, the World Health Organization [1] estimates that 1 in every 10 people will experience disabling hearing loss. In the United States, approximately 15% of adults (37.5 million) aged 18 and over report having some trouble hearing [2], and in the European Region, approximately 20% of the population (190 million) disclose having hearing loss or deafness [3]. This condition significantly impacts the quality of life for affected individuals, with communication and speech becoming the biggest challenges [4].

Hearing aids (HAs) and cochlear implants (CIs) are the primary treatment options for permanent sensorineural hearing loss [4]. Despite their benefits in improving speech perception [5,6] and quality of life [7], both devices suffer from low adoption rates due to perceived ineffectiveness, ongoing maintenance costs, lack of comfort, activity limits, and aesthetics [8,9]. A proposed solution to address some of these issues is a totally implantable auditory prosthesis (TIAP) in which all device components are surgically implanted. Fully implantable sensors like an accelerometer placed on the ossicles provide protection for external effects like wind noise, cerumen blockage, and water loading. In addition, a middle ear sensor automatically provides middle ear gain and pinna directionality cues, thereby enhancing acoustic performance over an externally housed sensor [10]. Although TIAPs have been developed and are currently under trial [11,12], researchers have stated that the main barrier to their wider adoption is the lack of a robust, broadband, fully implantable acoustic sensor [13,14]. In this paper, we seek to advance the development of a TIAP by providing concrete guidance on the location and orientation of a middle ear sensor.

Developing an implantable sensor that can rival external commercial microphones has proven to be a substantial undertaking [14]. In the literature, there are various examples of prototype implantable sensors, including those detecting acoustic vibrations directly, e.g., an implantable microphone [15,16], those detecting the resulting vibrations of the middle ear bones [17,18,19,20,21], and those utilizing the pressure changes within the inner ear, e.g., a pressure sensor [22]. However, none of the approaches in the literature can adequately achieve a performance over the entire operational frequency bandwidth of 100 Hz–8 kHz [23,24] that rivals external commercial microphones in small form factors. The accelerometer design used in this paper is based on the accelerometer performance presented by Hake et al. [25], which has the potential to address a gap in the literature on implantable sensors by meeting the required space constraints and the 20-phon noise level over the 100 Hz–8 kHz frequency range when placed on the umbo of the malleus.

To our knowledge, none of the prototype middle ear sensors in the literature have been optimized for their attachment within the middle ear cavity. As the ossicular chain does not vibrate in one simple manner for all frequencies [26], the frequency-dependent behavior and complex motion of the middle ear ossicles could significantly affect the performance of an accelerometer attached to these ossicles. Because umbo motion is the greatest [27], it is one of the first choices for the placement of a middle ear accelerometer. However, other locations on the ossicles may be preferred from a surgical standpoint. In particular, the short process of the incus is a location of interest as it is accessed by a mastoidectomy and used to identify the facial nerve, a step necessary in standard CI surgery [28].

In this study, we optimize the location and orientation of the accelerometer presented by Hake et al. [25] placed on the short process of the incus with respect to the minimum detectable sound pressure level (SPL). This optimization is made possible by the use of a finite element (FE) simulation of a human middle ear model developed and validated against experimental data by Sackmann et al. [29] and adapted by Burovikhin et al. [30]. The FE model is used to simulate the sound-induced rigid-body motion of the ossicles and the response of the sensor as a function of location and orientation. This placement location has not been studied previously, and no exhaustive optimization of this sort has been undertaken. Moreover, this optimization study provides us with insights regarding robustness to perturbations in terms of the orientation on the incus, which, in turn, provides guidance to surgeons for placing sensors. As a basis for comparison, these results on the incus are compared to the performance of the sensor when placed on the umbo.

## 2. Methods

### 2.1. Accelerometer Design and Sensitivity to Base Motion

**Figure 1:**
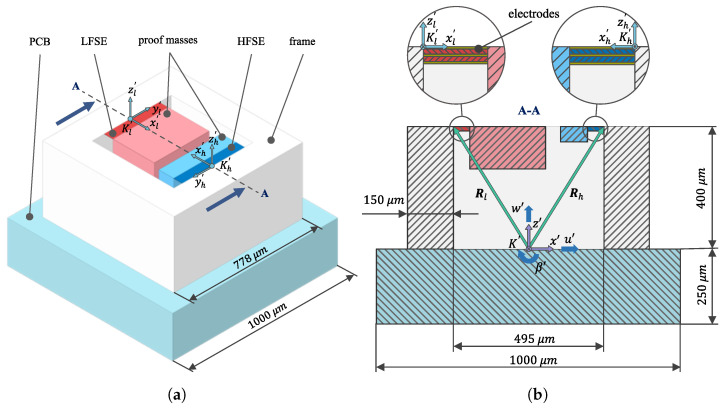
(**a**) The design of the accelerometer. (**b**) A cross-section view of the sensor in the A-A plane. The inset shows the piezoelectric material electrode stack of the bimorph structure as well as the local coordinate system for each sensing element.

**Figure 2:**
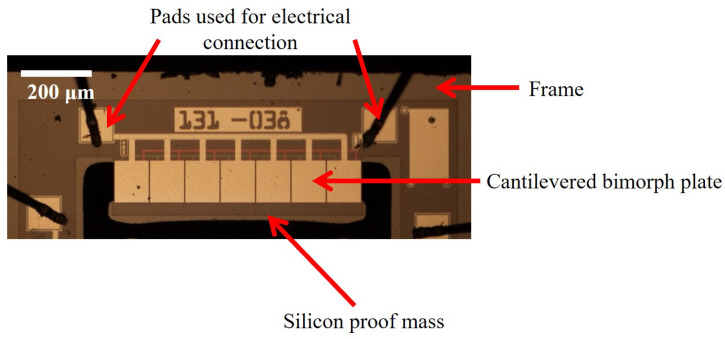
One sensing element from the fabricated accelerometer design presented in [31]. The full fabricated accelerometer design consisted of four electrically independent sensors within each frame.

In the present paper, the piezoelectric MEMS (micro-electro-mechanical system) accelerometer design from Hake et al. [25], shown in Figure 1, is used as the basis for our optimization study. A similar accelerometer design was fabricated by Hake et al. [31], and one sensing element from that design is shown in Figure 2. This fabricated accelerometer was used to experimentally validate the predictive model used throughout this paper. In the dual-bandwidth design, each MEMS die consists of two sensing elements: a low-frequency sensing element (LFSE, shown in red) that operates in the range from 100 Hz to 1.25 kHz, and a high-frequency sensing element (HFSE, shown in blue) that operates from 1.25 kHz to 8 kHz. The sensing elements comprise cantilevered bimorph plates, which are supported at their base by a silicon (Si) frame, shown in white, and are connected at their free end to a silicon proof mass. The sensing elements have lengths *L*, thicknesses $t_{b}=1$ μm (with 0.5 μm thick aluminum nitride (AlN) layers), and widths *b*. Three molybdenum (Mo) electrodes with thicknesses of 20 nm are deposited on the top, middle, and bottom of each bimorph, with the middle electrode grounded (see the inset of Figure 1b). The silicon proof masses have dimensions of lengths $L_{M}$ (in the local $x^{\prime }$-direction), thicknesses $t_{M}$, and widths $W_{M}$. All the dimensions are given in Table 1. The fabrication and design of these sensors are described by Hake [32] and Hake et al. [31].

The MEMS die is modeled as mounted on a base (e.g., a printed circuit board), as shown in blue in Figure 1, which can be used to interface with signal-conditioning electronics. In Figure 1b, the vectors $R_{l}$ and $R_{h}$ are the translations from the origin of the sensor base ($K^{\prime }$) to the LFSE origin $K_{l}^{\prime }$ and HFSE origin $K_{h}^{\prime }$, respectively. Since the Mo electrodes are thin, their effect on beam-bending mechanics is neglected in the model. Furthermore, since the piezoelectric plates are wider than they are thick, we use the plane–strain constitutive relationship. Thus, the AlN material properties used are density, $\rho_{AlN}=3.26\times 10^{3}$ (kg/m^3^); elastic compliance, measured under a constant electric field, $s_{11}=2.6\times 10^{-12}\left(m^{2}/N\right)$; the piezoelectric coefficient, $d_{31}=-2.5\times 10^{-12}\left(C/N\right)$; permittivity, measured under constant stress, $\epsilon_{33}^{T}=10.0\cdot 8.854\times 10^{-12}\left(F/m\right)$; and the piezoelectric loss tangent, tan(δ)=0.0015 [31]. Viscoelastic damping is included by defining a complex elastic modulus as $E^{*}=E\left(1+\eta \right)$, where η=0.06 is applied to each plate. Previous studies [25] indicate the robustness of this sensor to very loud sounds. A final packaged sensor design would require a protective lid (likely fabricated from titanium), which is not shown in Figure 1.

**Figure 3:**
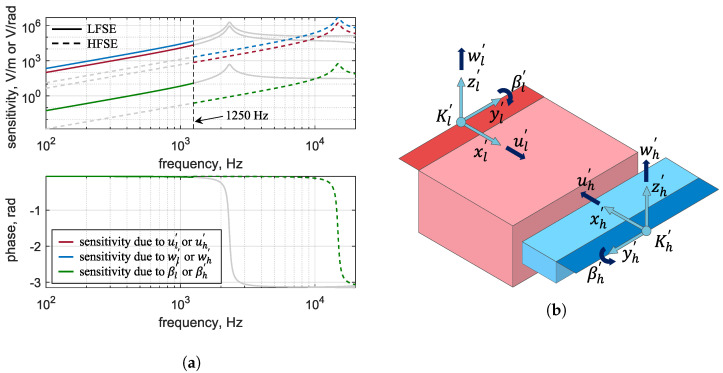
(**a**) The sensitivity curves and corresponding phase plots of each sensing element (LFSE and HFSE) expressed in V/m and V/rad, respectively. The greyed–out curves in each graph represent sensitivities outside the frequency bandwidths of each sensing element. The phase plots overlap, and hence, only $\beta_{l}$ and $\beta_{h}$ are visible. (**b**) The coordinate systems of each sensing element, $K_{l}^{\prime }$ and $K_{h}^{\prime }$, along with the positive sense of displacement and rotation, are shown relative to the sensor schematic.

We used the analytical model of the sensor presented by Hake et al. [31,33] to predict the voltage output due to motion at the origin ($K_{l}^{\prime }$ and $K_{h}^{\prime }$) of each piezoelectric bimorph plate. The coordinate directions associated with the sensor are defined in the inset of Figure 3. Based on symmetry arguments and our own finite element simulations of the sensor, we found that the voltage output is far more sensitive to the translational excitation in the *x*- and *z*-axes (designated as *u*, *w*) and to the rotational excitation around the *y*-axis (β) than in the other three degrees of freedom (*v*, α, and γ). Hence, these latter three degrees of freedom are not included in the analysis.

The sensitivity of each sensing element is defined as its voltage output normalized to the induced excitation (translational or rotational) at the origin of the cantilever. Figure 3a shows all the sensitivity curves for the LFSE and HFSE sensing elements derived in their corresponding coordinate systems, $K_{l}^{\prime }$ and $K_{h}^{\prime }$, which are shown in Figure 3b.

To accurately reflect the rotational and translational motion seen at $K_{l}^{\prime }$ and $K_{h}^{\prime }$, we first must relate the motion at each origin to the motion of the base ($K^{\prime }$, shown in Figure 1b). To do this, the motion of the sensor at a certain point in space *i*, in the accelerometer’s base coordinate system, $K^{\prime }$, is expressed by the displacement vector $q_{i}=\left[u_{i}^{\prime }\;v_{i}^{\prime }\;w_{i}^{\prime }\;\alpha_{i}^{\prime }\;\beta_{i}^{\prime }\;\gamma_{i}^{\prime }\right]$. To obtain the voltage produced by each sensing element due to the sensor’s *base* motion, the displacements $q_{i,l}$ and $q_{i,h}$ are calculated at the origins of $K_{l}^{\prime }$ and $K_{h}^{\prime }$, as follows:

(1) $$\begin{array}{c} \left[\begin{array}{c} u_{i,l}^{\prime }\\ v_{i,l}^{\prime }\\ w_{i,l}^{\prime } \end{array}\right]=R_{\mathrm{rot},l}\cdot R_{l}+\left[\begin{array}{c} u_{i}^{\prime }\\ v_{i}^{\prime }\\ w_{i}^{\prime } \end{array}\right]\;,\;\left[\begin{array}{c} u_{i,h}^{\prime }\\ v_{i,h}^{\prime }\\ w_{i,h}^{\prime } \end{array}\right]=R_{\mathrm{rot},h}\cdot R_{h}+\left[\begin{array}{c} u_{i}^{\prime }\\ v_{i}^{\prime }\\ w_{i}^{\prime } \end{array}\right] \end{array}$$

(2) $$\begin{array}{c} \left[\begin{array}{c} \alpha_{i,l}^{\prime }\\ \beta_{i,l}^{\prime }\\ \gamma_{i,l}^{\prime } \end{array}\right]=R_{\mathrm{rot},l}\cdot \left[\begin{array}{c} \alpha^{\prime }\\ \beta^{\prime }\\ \gamma^{\prime } \end{array}\right]\;,\;\left[\begin{array}{c} \alpha_{i,h}^{\prime }\\ \beta_{i,h}^{\prime }\\ \gamma_{i,h}^{\prime } \end{array}\right]=R_{\mathrm{rot},h}\cdot \left[\begin{array}{c} \alpha^{\prime }\\ \beta^{\prime }\\ \gamma^{\prime } \end{array}\right] \end{array}$$

where $R_{\mathrm{rot},l}$ and $R_{\mathrm{rot},h}$ are the rotation matrices for the transformation from the $K^{\prime }$-frame to the $K_{l}^{\prime }$-frame and $K_{h}^{\prime }$-frame, and $R_{l}$ and $R_{h}$ are the positional vectors from the origin of the $K^{\prime }$-frame to the origins of the $K_{l}^{\prime }$-frame and $K_{h}^{\prime }$-frame. The voltage output is calculated by multiplying the displacement vectors $q_{i,l}=\left[u_{i,l}^{\prime }\;v_{i,l}^{\prime }\;w_{i,l}^{\prime }\;\alpha_{i,l}^{\prime }\;\beta_{i,l}^{\prime }\;\gamma_{i,l}^{\prime }\right]$ and $q_{i,h}=\left[u_{i,h}^{\prime }\;v_{i,h}^{\prime }\;w_{i,h}^{\prime }\;\alpha_{i,h}^{\prime }\;\beta_{i,h}^{\prime }\;\gamma_{i,h}^{\prime }\right]$ with the corresponding sensitivity curves derived from the analytical model.

**Figure 4:**
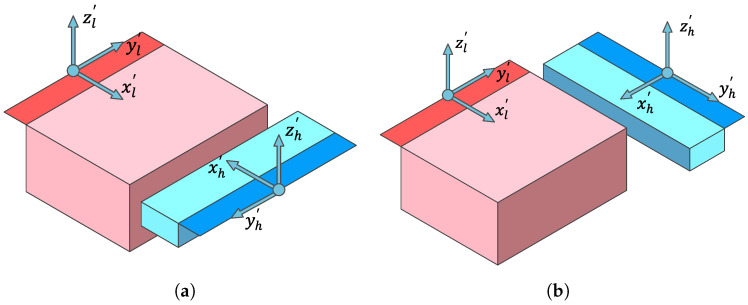
(**a**) configuration 1; (**b**) configuration 2.

In order to study the most general case of alignment for the two sensing elements, two different configurations are investigated, as shown in Figure 4a,b. In configuration 1, the sensing elements are opposite one another, while in configuration 2, the HFSE is rotated by 90° around its *z*-axis and placed perpendicular to the LFSE. In both configurations, the low-frequency axis system, $K_{l}^{\prime }$, is aligned with the frame system, $K^{\prime }$, as shown in Figure 1b. A reason to study these perpendicular orientations is to determine if significant differences in the sensitivity (and thereby the best device orientation) might arise. Sensitivity differences could arise due to a frequency-dependent change in the mode of the ossicular response to acoustic input, resulting in a shift in the dominant motion direction at the attachment point.

### 2.2. Middle Ear Model as a Virtual Testing Environment

**Figure 5:**
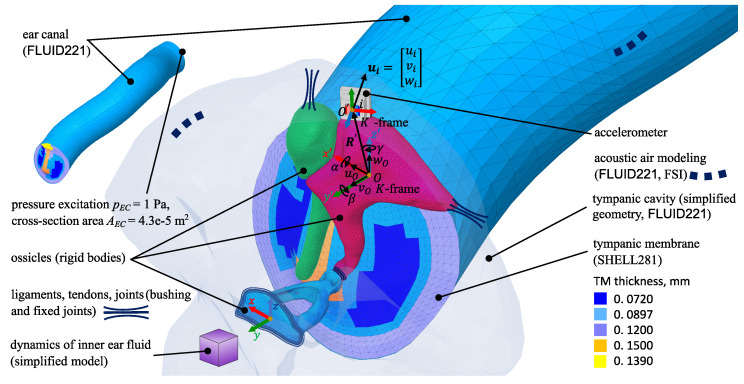
The finite element model of the middle ear [29,30].

In order to predict the translation and rotation at the base of the accelerometer (at the origin of the $K^{\prime }$ coordinate system), a finite element (FE) model of the middle ear was used to predict the motion of the ossicles in response to sound. We used ANSYS 2022R1 as the FE program to create the model shown in Figure 5. The geometry of the tympanic membrane (TM), the ossicles, the ear canal, and the tympanic cavity was reconstructed using micro-CT [29,34]. This model was originally developed by Sackmann et al. [29,34] /hland adapted by Burovikhin et al. [30]. Any changes implemented for the present study will be described in the overview of the model that follows.

The TM is divided into five regions. Each region has a constant thickness derived from the characteristic relative thickness distribution measured by van der Jeught et al. [35]. The five regions are represented by five different colors. The TM is meshed using second-order shell elements (Shell181). The translational degrees of freedom of the TM’s outer edge are fixed.

The ear canal and tympanic cavity are meshed with second-order Fluid221 tetrahedral elements. The aditus ad antrum and the air in the mastoid are modeled as a one-mass oscillator, which represents the oscillating air between the tympanic cavity and the mastoid and corresponds to a Helmholtz resonator (mass m=0.02 mg, stiffness c = 18 N/m, damping d=0.04 Ns/m). The coupling between the TM and the adjacent fluid volumes (ear canal and tympanic cavity) is implemented using a fluid–structure interaction (FSI) interface in ANSYS Mechanical. To define the FSI, continuity of fluid and structural displacement in the direction normal to the structure–fluid interface is enforced. The linearized Euler’s relation is used to relate the fluid pressure to the structural acceleration, while the fluid pressure acts as a load on the shell structures of the tympanic membrane.

The ear canal and tympanic cavity walls are considered rigid, which is the default boundary condition for acoustic bodies in ANSYS Mechanical. The geometry of the tympanic cavity is simplified by using plane side walls with an overall cavity of 0.63 mL, which fits within the expected range of 0.51–0.85 mL defined in [36,37]. Differing from the model used by Sackmann et al. [29,34], a 40 mm long ear canal is used in this study. In the model used by Sackmann et al. [29], a boundary condition with acoustic absorption elements is used to model an acoustic opening with a 2–3 mm diameter in the tympanic cavity. This opening replicates the facial recess access opening in the referenced temporal bone experiments. This opening is closed in the ANSYS model used for this study.

To model the ossicles, first, a modal analysis for each free ossicle is conducted in ANSYS using the following material properties: a Young’s modulus of $1.2\cdot 10^{10}$ N/m^2^; a Poisson’s ratio of 0.3; and a homogenized density of the compacta and spongiosa bone layers of 2400 kg/m^3^. Since the natural frequency of the first elastic deformation of the ossicles is above 30 kHz (malleus: 29.8 kHz; incus: 31.3 kHz; stapes: 51.5 kHz), the ossicles are modeled as rigid bodies. Thus, the elastic deformation of the ossicles is assumed to be negligible for a frequency range of up to 10 kHz, which is well above the present study’s upper-frequency limit of 8 kHz.

The malleus and incus each have six degrees of freedom. The stapes is constrained, allowing for a translational piston motion along the lateral–medial *y*-axis and two rotational (rocking) motions around the anterior–posterior *x*- and superior–inferior *z*-axes of the stapes coordinate system.

The ligaments, tendons, and joints are represented by massless generalized spring–damper elements, each with six uncoupled stiffnesses and damping properties. They are modeled as bushing joints with a multiple-point constraint formulation. The annular ligament model is simplified to only three decoupled stiffnesses and damping properties corresponding to the three degrees of freedom of the stapes. The dynamics of the inner ear are simplified to an overdamped one-mass oscillator, resulting in a cochlear impedance that has an almost flat spectrum [30].

The FE simulations provide a description of the ossicular motion. The motion from the FE model is normalized to the pressure applied at the inlet of the ear canal. We can then use these predictions to determine the velocity anywhere on the ossicles or for an accelerometer rigidly attached to the incus or malleus using the relations of rigid-body kinematics [38], a process that will be described after considering anatomical restrictions on the sensor placement.

### 2.3. Placement and Anatomical Restrictions

Before studying the effect of the middle ear location and the orientation of the accelerometer on the output voltage and inherent noise, we seek to identify surgical and anatomical limitations that will constrain possible locations. Due to the small size of the stapes [39,40], its lower vibration levels, and its relative inaccessibility, only the malleus and the incus are viable attachment points. On the malleus and incus, three locations are large enough to accommodate a sensor: the umbo of the malleus, the long process of the incus, and the short process of the incus. Of these, only the short process of the incus can be accessed through standard cochlear implant surgical procedures [28,41,42].

In typical cochlear implant surgery, the first step is a mastoidectomy, which involves removing the mastoid air cells from the mastoid bone located behind the pinna. During this step, it is necessary to open the aditus ad antrum to reveal the short process of the incus [43]. The short process is one of the standard landmarks used to identify the location of the facial nerve [28]. The second step is a posterior tympanotomy (facial recess approach), which involves further drilling into the mastoid to create a small opening into the middle ear space. The opening into the middle ear space is restricted by the position of the facial nerve and the chorda tympani nerve [28,41]. The posterior tympanotomy allows access to the distal long process of the incus and the round window; the latter is needed for cochlear implant surgery [41]. The size of the sensor to be implanted may require a larger opening than in standard cochlear implant surgery in order for the sensor to fit into the middle ear space. This is the case for the MED-EL (Med-El, Insbruck, Austria) Vibrant Soundbridge [44,45] middle ear implant. A large opening would also allow access to the umbo of the malleus, as we showed in our temporal bone experimentation [33]. However, a large opening increases the risks of facial paralysis and/or taste dysfunction [44,45].

The umbo of the malleus may also be accessed through a transcanal approach using a lower-risk procedure, which involves lifting the eardrum [46,47]. A posterior tympanotomy would still be required to access the sensor’s wiring and retrieve its output to ensure no wires are exposed externally in the ear canal.

Therefore, the short process of the incus presents an advantage as it does not require a posterior tympanotomy, and can be accessed only through a standard mastoidectomy with an extended antrotomy, which is a more straightforward and lower-risk procedure [45]. If a posterior tympanotomy is needed in the case of cochlear implant surgery, the opening into the middle ear space does not need to be enlarged as this would add unnecessary risk. In addition, there is ample room posterior to the short process for the routing of wires and other practical aspects.

While the vibrational signal at the umbo is greatest [27,48], implantation at this site involves additional surgical risks or a more complicated procedure (as discussed above). Thus, the short process of the incus holds great potential for sensor placement, but this region has not yet been extensively explored. Because of these factors, the search for the best location and optimal orientation of the accelerometer presented in [25] is restricted to the superior surface of the short process of the incus, from the posterior incudal ligament to the incudomalleolar joint. The results of the optimization on the incus will be compared to the umbo acceleration results for reference.

### 2.4. Finding the Optimal Position and Orientation for the Accelerometer

The FE formulation described in Section 2.2 is used to estimate the rigid-body motion of the incus. Assuming that the accelerometer package allows for nearly rigid incudal attachment, the translational and rotational displacements at any given point on the accelerometer can be derived using formulas from rigid-body kinematics, as we describe next. With this base motion predicted, the output voltage of the accelerometer due to the pressure force at the eardrum is computed as outlined in Section 2.1. This results in a prediction of the pressure sensitivity of the device (converting the accelerometer into a microphone).

The rigid-body motion of the incus consists of three translational components and three rotational components about a reference point *O* in the reference frame *K*, where *O* is located at the center of mass of the incus. The *K*-frame and the corresponding six rigid-body motion components are shown in Figure 5. The landmarks of the stapes are used to set up the coordinate system. The *x*-axis is parallel to the long axis of the stapes footplate with the anterior direction set as its positive direction, the *z*-axis is parallel to the short axis of the stapes footplate with the superior direction set as its positive direction, and the *y*-axis is orthogonal to the stapes footplate plane, forming a right-handed reference system.

In the *K*-frame, the three translational displacements of the origin are $u_{0}$, $w_{0}$, and $v_{0}$, and the three rotational displacements of the incus are α, β, and γ. The motion of the incus must be translated to the $K^{\prime }$-frame of the sensor in order to compute the output voltage. The motion in the $K^{\prime }$-frame is different from the motion of the ossicle to which the sensor is attached due to the height and orientation of the package. In other words, the titanium lid and any clamps used to attach the packaged sensor to the ossicle can be used to introduce spacing between the ossicle and the base of the MEMS accelerometer. The linearized 3D displacement components at any point $P_{i}$ on the accelerometer ($u_{i}$, $v_{i}$, and $w_{i}$) are related to the six rigid-body motion displacements of the incus as follows:

(3) $$u_{i}=u_{O}+\vartheta_{K}\times R^{\prime },$$

with

$$u_{i}=\left[\begin{array}{c} u_{i}\\ v_{i}\\ w_{i} \end{array}\right],u_{O}=\left[\begin{array}{c} u_{O}\\ v_{O}\\ w_{O} \end{array}\right],\vartheta_{K}=\left[\begin{array}{c} \alpha \\ \beta \\ \gamma \end{array}\right]\mathrm{and}\;R^{\prime }=\left[\begin{array}{c} x_{i}\\ y_{i}\\ z_{i} \end{array}\right],$$

where $R^{\prime }$ represents a fixed-body position vector pointing from the origin *O* to point $P_{i}$ in the *K*-frame.

Since the accelerometer can have any orientation in relation to the *K*-frame, a body-fixed frame $K^{\prime }$ has to be introduced; see Figure 1. To calculate the voltage produced by the sensing elements, the displacements at the accelerometer’s point $P_{i}$ and the rotational displacements, $\vartheta_{K}$, of the *K*-frame should be expressed in the accelerometer’s $K^{\prime }$-frame. With the help of the Cardan angles φ, ϑ, and ψ, the rotation matrix, $R_{KK^{\prime }}$, between the *K*-frame and the $K^{\prime }$-frame can be defined as

(4) $$R_{KK^{\prime }}=\left[\begin{array}{ccc} 1 & 0 & 0\\ 0 & \cos\varphi & -\sin\varphi \\ 0 & \sin\varphi & \cos\varphi \end{array}\right]\cdot \left[\begin{array}{ccc} \cos\vartheta & 0 & \sin\vartheta \\ 0 & 1 & 0\\ -\sin\vartheta & 0 & \cos\vartheta \end{array}\right]\cdot \left[\begin{array}{ccc} \cos\psi & -\sin\psi & 0\\ \sin\psi & \cos\psi & 0\\ 0 & 0 & 1 \end{array}\right].$$

Due to the orthonormality of the rotation matrix, $R_{KK^{\prime }}^{T}=R_{KK^{\prime }}^{-1}$, the following transformation rule can be applied:

(5) $$u_{i,K^{\prime }}=R_{KK^{\prime }}^{T}\cdot u_{i,K}\;\mathrm{and}\;\vartheta_{K^{\prime }}=R_{KK^{\prime }}^{T}\cdot \vartheta_{K},$$

whereby

(6) $$u_{i,K^{\prime }}=\left[\begin{array}{c} u_{i}^{\prime }\\ v_{i}^{\prime }\\ w_{i}^{\prime } \end{array}\right]\;\mathrm{and}\;\vartheta_{K^{\prime }}=\left[\begin{array}{c} \alpha^{\prime }\\ \beta^{\prime }\\ \gamma^{\prime } \end{array}\right].$$

The resulting rotational and translational displacements at point $P_{i}$ in the accelerometer’s $K^{\prime }$-frame are denoted with the vector $q={\left[u_{i}^{\prime }\;v_{i}^{\prime }\;w_{i}^{\prime }\;\alpha^{\prime }\;\beta^{\prime }\;\gamma^{\prime }\right]}^{T}$. The orientation of the accelerometer is determined by looping through the φ, ϑ, and ψ angles of the $K^{\prime }$-frame at each short process location point. The voltage produced by each sensing element is calculated by multiplying each element’s displacement vector q by its corresponding sensitivity curve; see Figure 3. Each voltage output is defined as the total voltage due to an input pressure excitation given in physical units of (V/Pa). Different accelerometer locations and orientations will lead to different sensing element voltage outputs. As previously stated, the main contributing voltage components are the voltages due to the translational excitation along the *x*- and *z*-axes and the rotational excitation around the *y*-axis. Using these FE simulations and subsequent coordinate transformations, we can estimate the total voltage output of the MEMS accelerometer with respect to the input acoustic pressure. This, then, is the acoustic pressure sensitivity of the device, denoted as *S*.

The pressure sensitivity is used to compute the input referred noise of the sensor due to the dielectric losses in the piezoelectric material. Using the Johnson noise equation, the spectral density of the sensor noise in 1 Hz bandwidths, denoted by $v_{noise}^{2}$, is given by

(7) $$v_{noise}^{2}=4\;k_{B}TR,$$

where $k_{B}$ is the Boltzmann constant, *T* is the temperature in Kelvin, and *R* is the real part of the electrical impedance of the piezoelectric layer (R=tanδ/ωC, with capacitance *C*, and the loss tangent of the piezoelectric material is tanδ). The input reference noise, $L_{N}$, computed in phon in accordance with the ISO standard [49] is

(8) $$L_{N}=40\log\left(B_{f}\right)+94\;\mathrm{phon},$$

where $B_{f}$ is

(9) $$B_{f}={\left[0.4\times 10^{\frac{nSPL+L_{U}}{10}-9}\right]}^{\alpha_{f}}-{\left[0.4\times 10^{\frac{T_{f}+L_{U}}{10}-9}\right]}^{\alpha_{f}}+0.005135$$

with $T_{f}$, $\alpha_{f}$, and $L_{U}$ beeing frequency-dependent parameters defined in the standard [49], and the noise-equivalent sound pressure level, nSPL, is

(10) $$nSPL=20\log\left(\frac{\sqrt{v_{noise}^{2}}}{S\cdot 20\mu \mathrm{Pa}}\right),$$

where the 1 Hz bandwidth of our computation is suppressed in Equation (10). Note that the sensor’s electronic noise is independent of the spatial orientation of the device.

We consider two criteria to evaluate the effect of location and orientation. Criterion 1 seeks to maximize the sum of the pressure sensitivity magnitudes, *S*, at two frequencies: 500 Hz for the LFSE and 7 kHz for the HFSE. Criterion 2 seeks to minimize the sum of the equivalent noise loudness levels, $L_{N}$, at these same two frequencies. We chose these two frequencies because they represent regions where the pressure sensitivity functions are relatively smooth, away from middle ear resonances or resonances in the sensitivity of either the LFSE or the HFSE; see Figure 3. Moreover, using two discrete frequencies simplifies the optimization. Each criterion is extremized by altering the accelerometer placement over the surface of the incus and rotating it over a complete sphere at each location.

**Figure 6:**
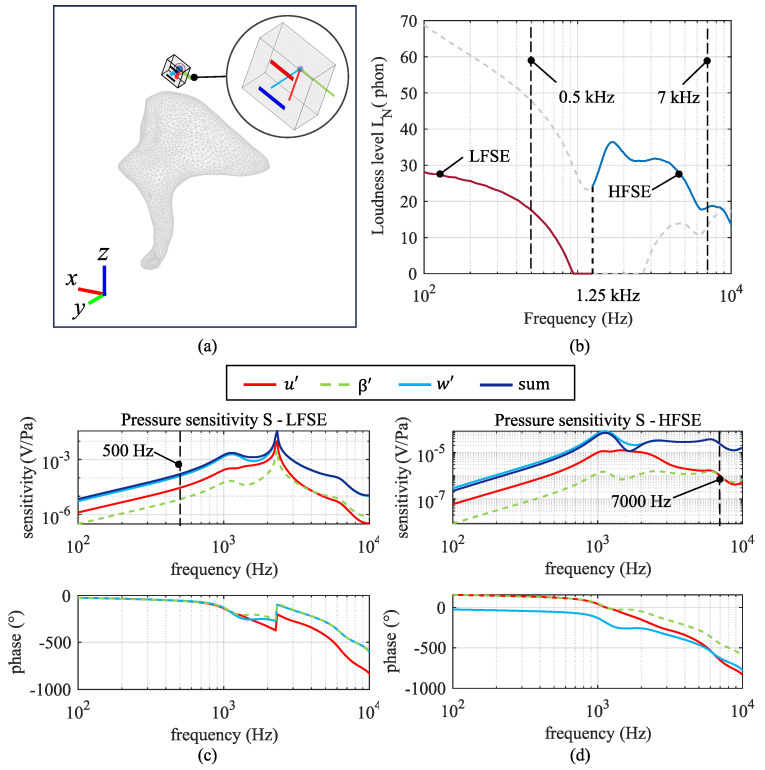
(**a**) The sensor positioned at a particular location (x=0.5 mm, y=4.4 mm, z=7.1 mm, φ=260°, ϑ=10°, ψ=70°) relative to the incus with configuration 1. The insert shows a close–up of the sensor together with the coordinate systems $K^{\prime }$, $K_{l}^{\prime }$ and $K_{h}^{\prime }$ (see Figure 1). (**b**) Equivalent loudness curves used to define the criteria for determining the optimal location and orientation of the accelerometer; (**c**) the pressure sensitivity produced by the LFSE at the shown position; (**d**) the pressure sensitivity produced by the HFSE at the shown position.

As an example, in Figure 6a, one possibility for the location and orientation of the accelerometer in configuration 1 is shown along with the incus (x=0.5 mm, y=4.4 mm, z=7.1 mm, φ=260°, ϑ=10°, ψ=70°). The inset shows a magnified version of the sensor and its $K^{\prime }$–system. Figure 6b shows the $L_{N}$ curves used for criterion 2. The dashed vertical lines indicate the frequencies at which the values are sampled for optimization. For both criteria, the first value is produced by the LFSE and sampled at 500 Hz, and the second is produced by the HFSE and sampled at 7 kHz. The data outside of the operational bandwidth of the sensing elements are represented by the grey curves. For criterion 1, we sample the total pressure sensitivity values, *S*, which are represented by the dark blue curves in Figure 6c,d. The red, green, and light blue curves represent the $u^{\prime }$, $\beta^{\prime }$, and $w^{\prime }$ sensitivities, respectively. The sampled values are summed up, and the sum is minimized for criterion 2 or maximized for criterion 1 by altering the accelerometer’s location and orientation.

## 3. Results

The best location and optimal orientation for configurations 1 and 2 of the accelerometer are chosen using the two previously stated criteria.

**Figure 7:**
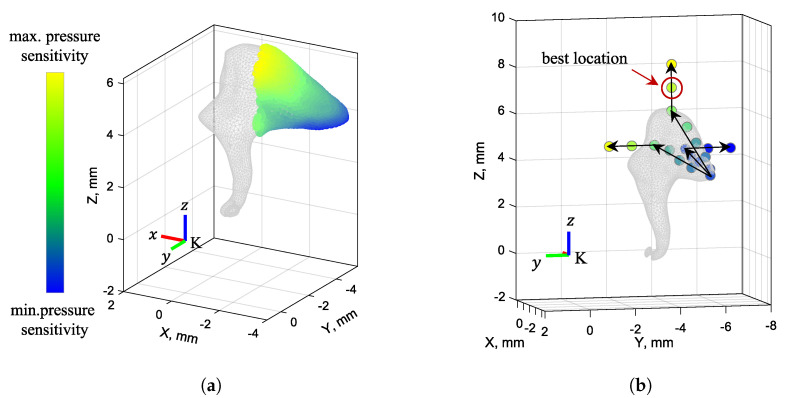
(**a**) The search space, whereby the locations with higher pressure sensitivity values are color–coded yellow and those with lower values are colored blue. (**b**) Best location shown along with the chosen surface points on the incus for a refined orientation search.

First, we searched for the highest pressure sensitivity by varying the sensor’s placement and orientation on the short process of the incus. These results are presented in Figure 7a. The initial search was performed for a high number of sample points on the incus (911 points) but with a low number of sensor orientations (13,824 orientations, sampling every 15°) to reduce the calculation time. This coarse sweep provided us with a good initial estimate of the locations where the pressure sensitivity is at its highest. Within the chosen search space, the highest pressure sensitivity values are reached near the incudomalleolar joint, whereas the lowest pressure sensitivity values are reached near the point where the posterior ligament is attached to the incus, as shown in Figure 7a.

After determining where the highest pressure sensitivity is reached, the sensor’s placement and orientation are varied for the two configurations shown in Figure 4. For a more thorough investigation, we used a higher number of sensor orientations, sampling every 5° (or 373,248 orientations), and a fewer number of sample locations, as shown in Figure 7b, on the lateral, medial, and superior sides of the incus surface, as well as off the incus surface at distances of 1 mm and 2 mm, representing possible sensor package distances from the incudal surface.

**Figure 8:**
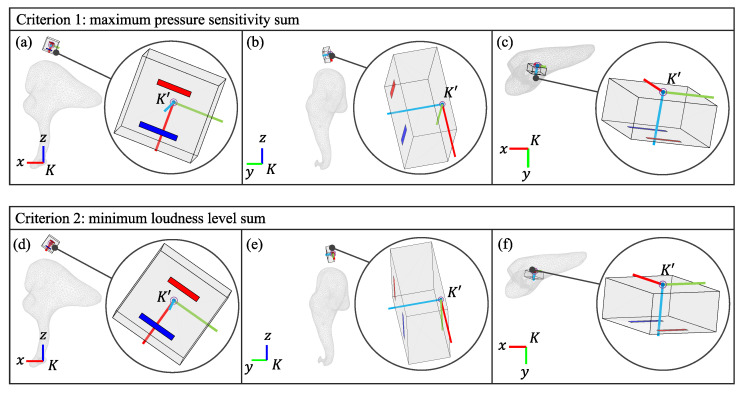
(**a**–**c**) Three views angles showing the optimal orientation of the accelerometer found through the maximum pressure sensitivity criterion at the best location for configuration 1. (**d**–**f**) Three views angles showing the optimal orientation of the accelerometer found through the minimum loudness level criterion at the best location for configuration 1.

**Figure 9:**
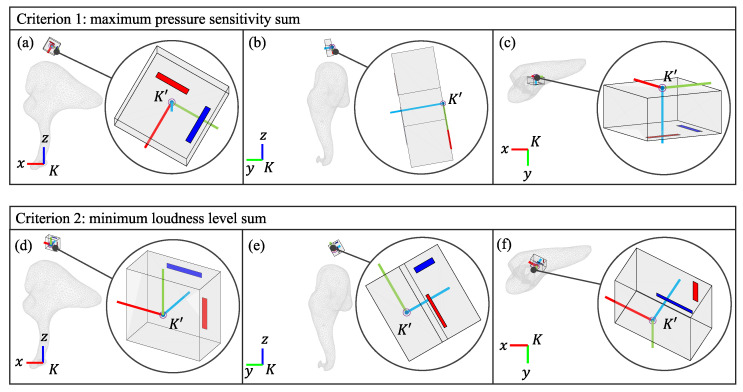
(**a**–**c**) Three views angles showing the optimal orientation of the accelerometer found through the maximum pressure sensitivity criterion at the best location for configuration 2. (**d**–**f**) Three views angles showing the optimal orientation of the accelerometer found through the minimum loudness level criterion at the best location for configuration 2.

From all the locations sampled on the short process of the incus, the best point for both criteria is near the incudomalleolar joint in the superior direction (the global *z*-direction), located roughly at a distance of 1 mm from the bone’s surface, as shown in Figure 7b. The optimal placement and orientation for the accelerometer based on the two criteria for configurations 1 and 2 are shown in Figure 8 and Figure 9. For each of the two criteria, three different views are shown relating the global coordinate system, *K*, to the accelerometer’s coordinate system, $K^{\prime }$. While the optimal location for both criteria is the same for both configurations, for the optimal orientation, configurations 1 and 2 are different. However, for both configurations, the orientations of the z’-axis are similar. Table 2 provides the numerical values for the best location and orientation found for configurations 1 and 2 for both criteria.

**Figure 10:**
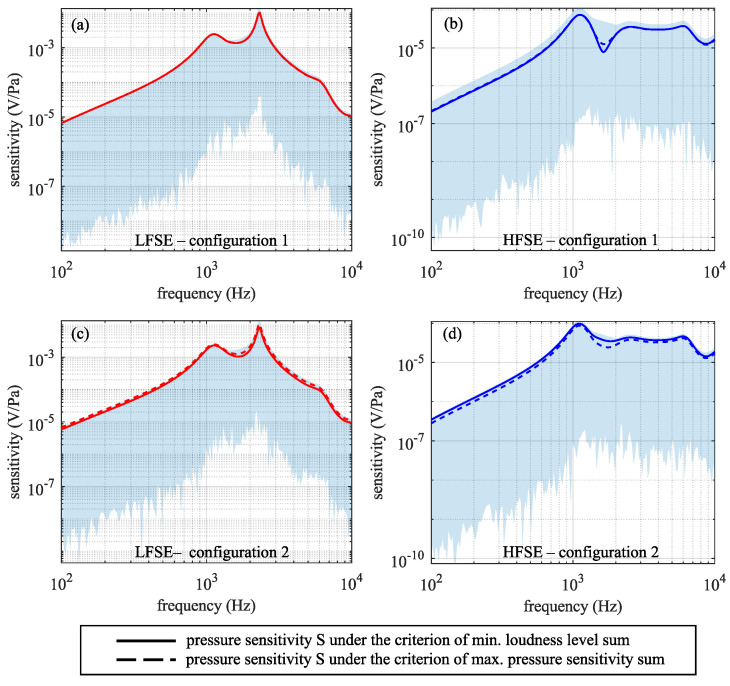
The pressure sensitivity produced by the LFSE (in red) and HFSE (in blue) at their best locations and optimal orientations as determined by each criterion. The plots are log–log, with each tic mark on the ordinate representing an order of magnitude change. (**a**,**b**) Configuration 1. (**c**,**d**) Configuration 2.

For each criterion, Figure 10 shows the pressure sensitivity frequency response produced by the LFSE and HFSE at their best locations and optimal orientations using a solid curve for critierion 1 and a dashed curve for criterion 2. The shaded areas represent the pressure sensitivity frequency response curves for all the other orientations. The sensitivity is seen to vary dramatically with orientation in the shaded region, but the two optimality criteria yield similar sensitivity results.

**Figure 11:**
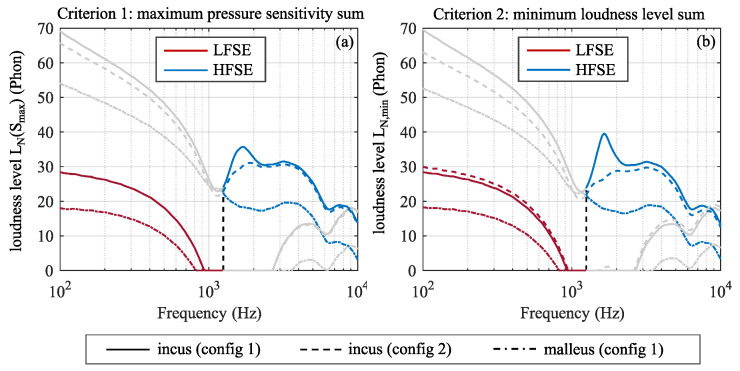
The comparison between configurations 1 and 2 as well as the placement of the accelerometer on the malleus. (**a**) Loudness level for criterion 1, the maximum pressure sensitivity. (**b**) Loudness level for criterion 2, the minimum loudness level.

We also compared the equivalent loudness levels, $L_{N}$ (Equation (8)), for the optimized designs found using criterion 1 and 2 in Figure 11. In the same plot, $L_{N}$ for the accelerometer in configuration 1 placed on the umbo of the malleus is shown for reference, since this location experiences the highest acceleration in the middle ear.

## 4. Discussion

As Figure 10 shows, both criteria yield similar sensitivities. Each criterion incorporates an average of factors (either of *S* or $L_{N}$) in the low-frequency and high-frequency bands. The maximum pressure sensitivity sum criterion gravitates towards finding the best orientation for the LFSE at the expense of the HFSE. This is indicated by the solid red curves being slightly lower than the dashed red curves in Figure 10a,c. This occurs because the LFSE produces a much higher pressure sensitivity at 500 Hz than the HFSE at 7 kHz. Thus, the LFSE makes a larger contribution to the sum than the HFSE, and, as the algorithm maximizes the sum, it favors the LFSE. In contrast, the minimum equal loudness sum criterion tends to favor the HFSE as it produces a lower equal loudness value at 7 kHz than the LFSE at 500 Hz, and the algorithm searches for the minimum sum. This is indicated by the solid blue curves being slightly higher than the dashed blue curves in Figure 10b,d.

The optimal orientations for both the maximum pressure sensitivity and the minimum loudness level criteria under both configuration 1 and configuration 2 result in the accelerometer’s $z^{\prime }$-axis in the $K^{\prime }$-frame pointing in the same direction as the global *y*-axis in the *K*-frame (see Figure 8 and Figure 9 and Table 2). The accelerometer under consideration has maximum sensitivity in response to translational excitation along the $z^{\prime }$-axis ($K^{\prime }$-frame). Since the incus principally performs a rocking motion around the global *x*-axis (*K*-frame) in response to acoustic input, it makes sense that the accelerometer’s optimal $z^{\prime }$-axis ($K^{\prime }$-frame) points to the global *y*-axis (*K*-frame), converting that rotation about the *x*-axis into a translation. Furthermore, we found that orientations close to this optimal result resulted in pressure sensitivities similar to the optimal values. By rotating the sensor 360° about the $z^{\prime }$-axis while maintaining the optimal $z^{\prime }$-axis orientation we found that criterion 1 changed by less than 1 dB. Hence, this design is relatively insensitive to rotations about the optimal $z^{\prime }$-axis alignment (see Table 2). We also rotated the device by adding ±10° to the optimal value of the angle $\varphi =260^{{}^{\circ}}$, the rotation angle about the global *x*-axis, and found that criterion 1 also changed by less than 1 dB (and this was true over all frequencies, except for a narrow range around 1.2 kHz–1.8 kHz, where a 4 dB change could be seen). Overall, this result provides direct guidance for a surgeon, as there is flexibility in orienting the device provided the sensor’s $z^{\prime }$-axis is oriented in this medial direction at a location near to the incudomalleolar joint.

There are minimal differences in the accelerometer’s performance between configuration 1 and configuration 2, as is evident from the curves shown in Figure 11, except for the frequency range between 1.2 kHz and 3 kHz, where configuration 2 shows better performance by 5–10 phon due to there being a deeper dip in the sensitivity of configuration 1 (Figure 10). Hence, configuration 2 appears to be more robust to this dip in sensitivity. Note that this dip may be unique to this particular middle ear. The performance is enhanced by roughly 10 dB if the accelerometer is attached to the umbo of the malleus, which is expected as the accelerations at that location are much higher than at any point on the short process of the incus. However, as addressed previously, it is less advantageous to place the accelerometer on the malleus as more complex surgery is required as compared to the posterior surface of the incus.

Lastly, there are two main aspects of this study that have not been fully investigated. One is the influence of the accelerometer’s mass on the overall dynamics of the system. It is expected that the larger the accelerometer, the more effect it will have on the system. According to our preliminary calculations, the accelerometer’s mass, including circuitry and packaging (15 mg), is much smaller than the mass of the ossicular chain, and thus, it will not have a large effect on the system’s performance. These calculations were conducted using the FE middle ear model, in which a point mass was used to represent the accelerometer and was rigidly attached to the incus at the best location determined in the present study. Gan et al. [50] conducted experiments on 17 temporal bones to study the effect on residual hearing due to mass-loading the ossicular chain. They found that even a weight of 22.5 mg can result in a 1.3 dB decrease in stapes displacement at low frequencies (250–750 Hz) and, on average, a 2.6 dB decrease across high frequencies (1000–8000 Hz). Thus, the accelerometer’s exact mass-loading effect on the short process of the incus should be further investigated.

The second aspect is the rigid connection of the accelerometer to the incus. In reality, this attachment will utilize a clip with a certain stiffness. The more compliant this connection is, the more effect the accelerometer’s mass will have on the system’s performance. The exact influence of the mass and accelerometer’s rigid connection on the overall system warrants further investigation.

## 5. Conclusions

In this study, a method for determining the best placement and optimal orientation of the piezoelectric accelerometer attached to the short process of the incus is developed. The best location is determined to be near the incudomalleolar joint, 1 mm away from the surface of the incus in the superior direction. This placement meets the anatomical limitations associated with this location and a posterior tympanotomy. It also takes into account the approximate dimensions of the clip or fixturing that will be used to attach the accelerometer to the incus. The optimal orientation of the sensor at the determined location is obtained using two criteria, the maximum pressure sensitivity sum and minimum loudness level sum. Both criteria result in the orientation of the accelerometer where the *z*-axis points towards the stapes’ footplate and the *y*-axis points along the short process of the incus towards the posterior ligament. We performed this optimization for one accelerometer design layout (as shown in Figure 1). The method used in this paper could also be used to optimize the sensor design itself (rather than keeping its design static). In this case, in addition to parameterizing the location of the sensor and its orientation, the physical dimensions of the sensor’s components and their material properties can also be optimized. The influence of the accelerometer’s mass and its connection to the incus should be investigated in more detail. Finally, fabrication and testing of the sensor attached to the incus remain important for future work.

## Figures and Tables

**Table 1:** Sensor design dimensions from Hake et al. [25].

Property	LFSE	HFSE
$t_{M}$ (μm)	140	50
$L_{M}$ (μm)	250	90
*L* (μm)	53	52
*b*, $W_{M}$ (μm)	333	378
$t_{b}$ (μm)	1	1

**Table 2:** Best location and orientation parameters for configurations 1 and 2. The orientation of the sensor depends on the configuration and optimization criteria, while the optimal location of the origin of the accelerometer is the same for all four cases.

Configuration	Criterion	φ	ϑ	ψ	Location [x, y, z]
1	Max. voltage sum	260°	10°	70°	$\left[\begin{array}{c} -0.5\;\mathrm{mm}\\ -4.4\;\mathrm{mm}\\ 7.1\;\mathrm{mm} \end{array}\right]$
	Min. loudness level	260°	5°	55°	
2	Max. voltage sum	260°	0°	60°	$\left[\begin{array}{c} -0.5\;\mathrm{mm}\\ -4.4\;\mathrm{mm}\\ 7.1\;\mathrm{mm} \end{array}\right]$
	Min. loudness level	60°	330°	0°	

## Data Availability

Data are available upon request from the corresponding author.
